# Distribution of ciliary adaptor proteins tubby and TULP3 in the organ of Corti

**DOI:** 10.3389/fnins.2023.1162937

**Published:** 2023-04-18

**Authors:** Laura A. Lindner, Dennis Derstroff, Dominik Oliver, Katrin Reimann

**Affiliations:** ^1^Department of Otorhinolaryngology, Head and Neck Surgery, University Hospital Marburg, Philipps-University Marburg, Marburg, Germany; ^2^Department of Neurophysiology, Institute of Physiology and Pathophysiology, Philipps-University Marburg, Marburg, Germany

**Keywords:** phosphoinositides, tubby, TULP3, hair cells, organ of Corti, immunohistochemistry

## Abstract

Tubby-like proteins are membrane-associated adaptors that mediate directional trafficking into primary cilia. In inner ear sensory epithelia, cilia—including the hair cell’s kinocilium—play important roles as organizers of polarity, tissue architecture and cellular function. However, auditory dysfunction in tubby mutant mice was recently found to be related to a non-ciliary function of tubby, the organization of a protein complex in sensory hair bundles of auditory outer hair cells (OHCs). Targeting of signaling components into cilia in the cochlea might therefore rather rely on closely related tubby-like proteins (TULPs). In this study, we compared cellular and subcellular localization of tubby and TULP3 in the mouse inner ear sensory organs. Immunofluorescence microscopy confirmed the previously reported highly selective localization of tubby in the stereocilia tips of OHCs and revealed a previously unnoticed transient localization to kinocilia during early postnatal development. TULP3 was detected in the organ of Corti and vestibular sensory epithelium, where it displayed a complex spatiotemporal pattern. TULP3 localized to kinocilia of cochlear and vestibular hair cells in early postnatal development but disappeared subsequently before the onset of hearing. This pattern suggested a role in targeting ciliary components into kinocilia, possibly related to the developmental processes that shape the sensory epithelia. Concurrent with loss from kinocilia, pronounced TULP3 immunolabeling progressively appeared at microtubule bundles in non-sensory Pillar (PCs) and Deiters cells (DC). This subcellular localization may indicate a novel function of TULP proteins associated with the formation or regulation of microtubule-based cellular structures.

## 1. Introduction

Hearing and balance are mediated by epithelial mechanosensory hair cells (Hudspeth, 2014). Mechanoelectrical transduction (MET) takes place within an apical bundle of protrusions, the hair bundle, consisting of multiple actin-based stiff stereocilia, and a single true (i.e., tubulin-based) cilium, termed kinocilium. Although in mammalian auditory hair cells of the cochlea (but not in the vestibular hair cells) the kinocilium degenerates before onset of hearing and is not required for MET by the sensory cells (Elliott et al., 2018), it has essential roles in development of the sensory epithelium and the hair cells, namely the establishment of hair bundle structure (Sharkova et al., 2023) and planar cell polarity (Deans, 2021).

In addition to hair cell kinocilia, supporting cells of the sensory epithelium also feature primary cilia at their apical surface. Much less is known about the function of supporting cell cilia in the inner ear (Jones and Chen, 2008); however, based on extensive work on various other epithelial organs where disruption of primary cilia leads to disturbed structure and function (‘ciliopathies’) (Focșa et al., 2021), it seems likely that supporting cell cilia have roles in development and maintenance of the sensory inner ear epithelia as well.

Primary cilia function as organelles and signaling hubs for sensing external stimuli. For this purpose, their membrane is enriched in receptors, including G-protein-coupled receptors and components of the respective downstream signal transduction cascades. Targeting and transport of these components into and within cilia is mediated by an elaborated intraflagellar transport (IFT) machinery (Picariello et al., 2019). Entry into the ciliary compartment is restricted by a proximal transition zone, requiring dedicated mechanisms that translocate dedicated cargo into the cilia. For some cargo including G protein-coupled receptors (GPCRs) this critical transport step involves members of the tubby protein family (tubby and tubby-like proteins, TULPs) (Badgandi et al., 2017). Briefly, these proteins are adaptors that bind to the cargo as well as the IFT-A complex, thereby favoring cargo transport into the cilium (Hong et al., 2021). Moreover, tubby/TULPs are characterized by a highly conserved carboxy-terminal domain, that binds to the signaling membrane lipid phosphatidylinositol-4,5-bisphosphate (PI(4,5)P_2_) (Boggon et al., 1999; Santagata et al., 2001; Thallmair et al., 2022). PI(4,5)P_2_ interaction contributes to directional import into cilia, since tight binding to the membrane favors formation of the tubby/TULP-cargo-IFT complex in the extraciliary membrane, which is rich in PI(4,5)P_2_. After reaching the flagellar membrane compartment, the complex is destabilized because the ciliary membrane contains no or little PI(4,5)P_2_, effectively completing cargo delivery to its destination (Dyson et al., 2017).

*Tubby* mice exhibit obesity, retinal degeneration, and hearing loss (Kleyn et al., 1996; Kong et al., 2009). So far, these phenotypes have largely been attributed to defects in primary cilia, i.e., they are considered as ciliopathies (Quinlan et al., 2008). Surprisingly, in the cochlea tubby was recently found to be localized exclusively at the tips of outer hair cell (OHC) stereocilia (Han et al., 2020), but not in kinocilia of cochlear and vestibular hair cells or in primary cilia of supporting cells. Moreover, tubby is required for proper localization of stereocilin (STRC), an essential component of the horizontal top connectors, connecting stereocilia of each row with each other and of the tectorial membrane-attachment crowns that connect the tallest row of stereocilia with the tectorial membrane (Han et al., 2020). Thus, tubby fulfills a non-canonical function in organizing a protein complex in the actin-based hair bundle rather than mediating ciliary trafficking. We therefore considered the possibility that a tubby homolog might adopt ciliary function, especially in hair cell kinocilia. TULP3 is the phylogenetically closest tubby homolog (Nishina et al., 1998) and has functional redundancy with tubby in mediating entrance of G-protein coupled receptors into primary cilia (Mukhopadhyay et al., 2010). It is expressed ubiquitously in the mouse embryo and is essential for normal embryonic development (Ikeda et al., 2001). Accordingly, TULP3-knockout mice die by embryonic day 14.5 with various abnormalities such as impaired neural tube closure, exencephaly and craniofacial cleft (Ikeda et al., 2001). TULP3 expression in the inner ear has not been described yet.

Here, we examined and compared expression and localization of TULP3 and tubby in the inner ear of postnatal mice using immune-fluorescence microscopy. Antibody staining confirmed the highly specific expression of tubby at stereociliary tips of mature OHCs, but we additionally observed localization to kinocilia and primary cilia in the developing cochlea. TULP3 localized to kinocilia of cochlear and vestibular hair cells in the neonatal inner ear, but expression was downregulated soon after birth. This localization pattern in hair cells, which is complementary to tubby, suggests a role of TULP3 in ciliary function during hair cell development. Moreover, TULP3 was found in supporting cells of the organ of Corti, with pronounced developmental upregulation. TULP3 localized to prominent tubulin-rich cytoskeletal structures, suggesting a previously unknown role in organizing or regulating cellular tubulin bundles.

## 2. Materials and methods

### 2.1. Animals

For the whole-mount preparations we used C57Bl/6 N wild type mice of both sexes. The age of the mice varied between postnatal day 0 (P0) and P30. This provided us with the opportunity of visualizing possible age-related expression differences. The samples were dissected from mice at different postnatal stages. The handling of the mice was according to the federal/institutional guidelines and approved by the local government.

### 2.2. Tissue collection

For whole-mount preparations, all mice were euthanized using isoflurane with a following decapitation. After removing the cochlea from the temporal bone and creating a small hole in the apical part of the cochlea, the tissue was fixed in 4% PFA. First, a 1 ml syringe with a 0.45 mm x 10 mm needle was used to flush the cochlea with the fixation. Therefor the needle was inserted into the oval and the round window and about 0.1 ml of the PFA injected into the cochlea. Next, the cochlea was transferred into a well, filled with the PFA, for 90 min at 4°C. After, the cochlea case was removed and the organ of corti dissected from the modiolus, the stria vascularis and the tectorial membrane. The mouse vestibular system was dissected as described in Cunningham (2006). The tissue was then used for whole-mount immunostaining and later mounted on Superfrost microscope slides.

### 2.3. Immunohistochemistry

Immunofluorescence staining of the whole-mount cochlea and utricle was performed as previously described (Dierich et al., 2019) with a few modifications. The tissue was transferred to 24 well plates and then blocked and permeabilized for 1 h at room temperature with 5% bovine serum albumin (BSA)/0.1% Triton X-100 in 1x Phosphate buffered saline (PBS). The samples were then incubated overnight, at 4°C (diluted in 5% BSA) with the following primary antibodies: rabbit-anti-tubby (Proteintech Group, 17928-1-AP; 1:100), rabbit-anti-TULP3 (Proteintech Group, 13637-1-AP; 1:100), mouse-anti-Acetylated-α-Tubulin (Proteintech, 66200-1-Ig; 1:400), and incubated over night at 4°C. The tissues were after rinsed with 1× PBS and incubated for 90 min at room temperature with a species appropriate secondary antibody, coupled to Alexa Fluor dyes (Thermo Scientific). If phalloidin was included in the staining, it was added to the secondary antibody for the last 30 min of the incubation time (anti-phalloidin-iFluor 633; Abcam, ab176758; 1:200). The tissues were rinsed again with 1× PBS. Nuclei were stained with 2 μg/mL 4′,6-Diamidine-2′-phenylindole 214 dihydrochloride (DAPI, Sigma-Aldrich, D9542) by addition with the second rinse. An antifade mounting medium was used to mount the tissue on Superfrost microscope slides (Thermo Scientific, P36961: Prolong Diamond Antifade mountant).

### 2.4. Cell culture & transfection

Chinese hamster ovary cells (CHO) were seeded on cover slips in glass bottom dishes and cultured using MEM (minimum essential medium) Alpha medium (Gibco, Thermo fisher Scientific, Waltham, USA) containing 10% fetal bovine serum, 1% penicillin and 1% streptomycin. The cells were kept at 37°C and 5% CO_2_. One day after seeding, cells were transfected using the JetPEI DNA transfection reagent (Polplus-transfection, Illkirch-Graffenstaden, France). Immunocytochemical stainings were perfomed 2 days after transfection.

### 2.5. Molecular biology

The following constructs were used for transfection & expression in CHO cells: Mouse full length Tubby in pEGFP-C1 (self-made) and human full length Tulp3 in pGLAP1 (gift from Mukhopadhyay Lab).

### 2.6. Immunocytochemistry

To evaluate the antibody specificity of the primary antibodies rabbit-anti-tubby (Proteintech Group, 17928-1-AP; 1:400) and rabbit-anti-TULP3 (Proteintech Group, 13637-1-AP; 1:400), CHO cells were transfected to express EGFP-tagged full length Tubby and Tulp3, respectively. Untransfected CHO cells were used as negative controls. The coverslips carrying the cells were transferred into a new 24-Well plate containing 4% PFA diluted in 1× PBS and incubated for 8 min at RT darkened. After removal of the PFA, the permeabilization buffer (0.1% Triton X-100 in 1× PBS) was added and incubated for 8 min at RT darkened. Cells were washed three times using a washing buffer (0.2% BSA in 1× PBS) for 10 min shaking at RT darkened. Primary antibodies were diluted in 2.5% BSA in 1× PBS and incubated for 60 min at RT darkened. After second washing of the cells, secondary antibody was diluted in 2.5% BSA in 1× PBS as well and incubated for 70 min darkened at RT. After final washing coverslips were mounted using the antifade mounting medium. The respective results are shown in Supplementary Figure 1.

### 2.7. Statistics

The Pearson correlation coefficient r was calculated using the Coloc 2 ImageJ plugin^1^. The distribution of the calculated Pearson coefficients r is presented as a dot plot in Supplementary Figure 2. The dot plot was created using the Igor Pro software (Version 8.04).

### 2.8. Confocal microscopy and data

Confocal imaging was performed on an upright LSM 710 Axio Examiner Z1, using a 40×/1,3 oil immersion objective. Data were acquired as z-stacks. Each staining was replicated at least 3 times. Representative images are shown as Z-projections.

## 3. Results

### 3.1. Developmental localization of tubby in hair bundles and primary cilia

**Figure 1:**
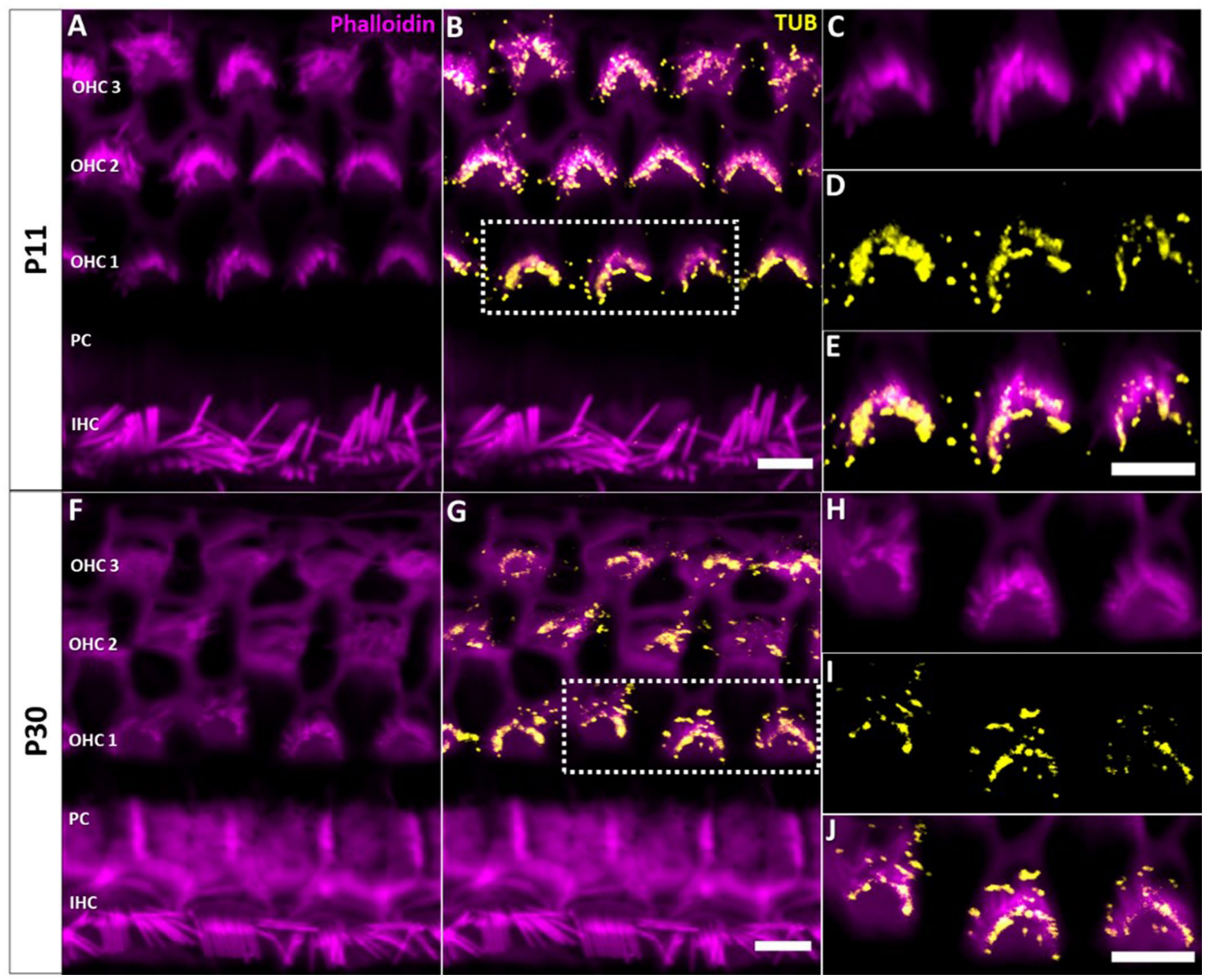
Localization of tubby immunoreactivity in the tips of OHC stereocilia. Whole mount immunohistochemical staining of Tubby (yellow) with Phalloidin (staining actin, magenta) in the mouse organ of corti. **(A,B)** P11, **(F,G)** P30. Strong immunoreactivity of tubby is present in the distal and basal parts of OHC stereocilia, corresponding to the horizontal tip connectors (HTC) and tectorial membrane attachment-crowns (TM-AC). **(C−E,H−J)** Close-up of identical staining at P11, i.e., P30. Scale bar 5 μm.

**Figure 2:**
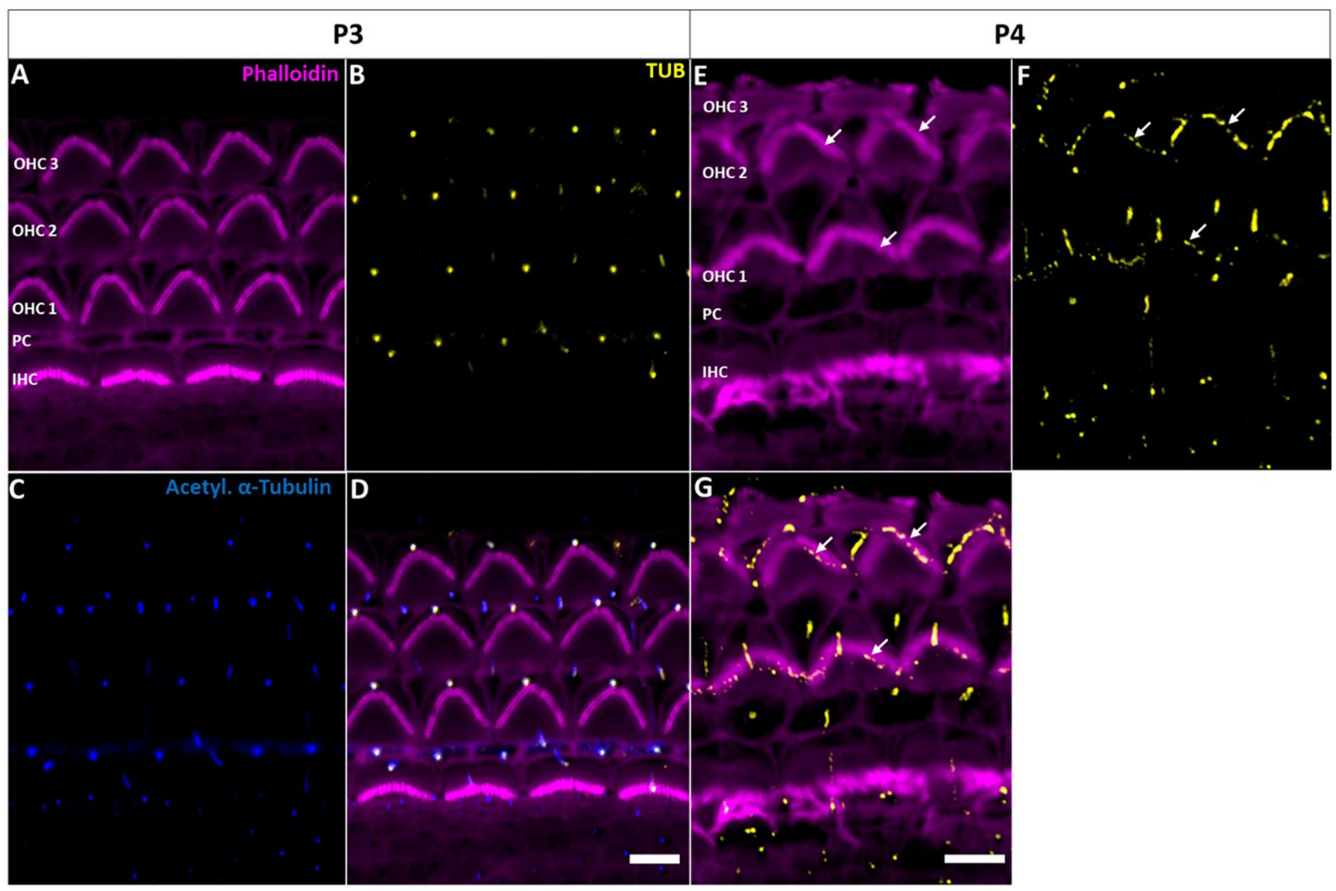
Tubby localization to hair cell kinocilia and supporting cell primary cilia in the early postnatal cochlea. **(A−D)** P3 whole-mount immunohistochemical staining of Tubby (yellow), Phalloidin (staining actin, magenta) and acetylated α-tubulin (blue) in the mouse organ of corti. Tubby is localized to the IHC and OHC kinocilia, while it is additionally localized to ad subset supporting cell primary cilia. **(E−G)** P4 whole-mount immunohistochemistry staining of Tubby (yellow) and Phalloidin (staining actin, magenta). Tubby is localized to hair cell kinocilia and a subset of supporting cell primary cilia (big arrows) but is additionally localized to the top of the tallest row of outer hair cells stereocilia (small arrows) Scale bar 5 μm.

Confocal microscopy of whole-mount preparations of the mouse organ of Corti detected tubby immunoreactivity selectively at the tips of stereocilia of developing (postnatal day P11, Figures 1A–E) and developmentally mature OHCs (P30, Figures 1F–J), but not in IHC hair bundles at either age. As described previously (Han et al., 2020), the localization to OHC stereocilia only developed during postnatal development. Labelling was undetectable at P3 (Figures 2A–D) and weak signals at the stereocilia tips first appeared at P4 (Figures 2E–G).

In early postnatal developmental stages (P3, P4), we additionally observed a single distinct tubby-positive structure at the apical surface of hair cells and supporting cells including Deiters’ and pillar cells (Figure 2). Colocalization with acetylated a-tubulin (Figures 2A–D) identified these structures as the kinocilia of OHCs and IHCs, and as primary cilia of supporting cells, respectively (Supplementary Figure 2). At P11, when cochlear hair cell kinocilia are degenerated, these tubby-positive structures were no longer detectable (Figures 1A–D). Similarly, labeling of cilia was no longer observed on the supporting cell surface (Figure 1). Thus, tubby localization to cilia developmentally precedes relocation to stereocilia tips in OHCs. While this spatiotemporal developmental pattern is consistent with the previously described function of tubby in organizing attachment complexes in OHC stereocilia (Han et al., 2020), tubby may additionally be involved in the function of primary cilia in sensory hair cells and non-sensory supporting cells during development.

### 3.2. TULP3 is localized to cochlear and vestibular hair cell kinocilia shortly after birth

Given both ciliary and non-ciliary localization of tubby in the sensory epithelium we wondered whether tubby homologs (TULPs) may show a similar or complementary pattern of localization. We focused on the closest related homolog TULP3, which functions in overlapping cellular processes with tubby, i.e., directional ciliary trafficking of GPCRs in neurons and retinal photoreceptors (Ikeda et al., 2002; Sun et al., 2012). Indeed, we observed robust immunoreactivity against TULP3 in the cochlea, revealing a complex spatiotemporal developmental pattern of expression and localization.

**Figure 3:**
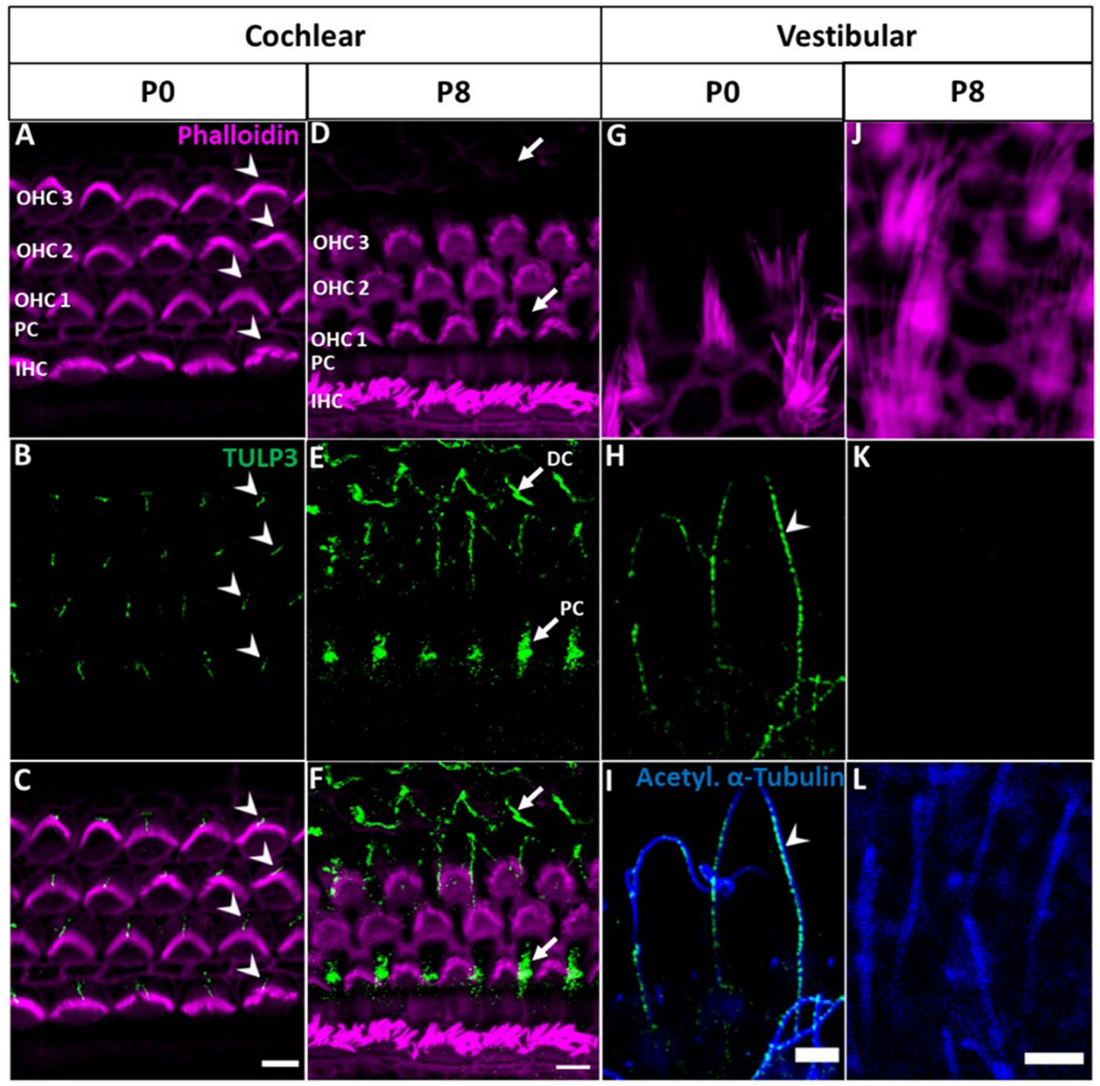
TULP3 immunoreactivity in the cochlear and vestibular epithelium of newborn mice compared with P8 mice. **(A**–**L)** P0 and P8 whole-mount immunohistochemical staining of TULP3 (green), Phalloidin (staining actin, magenta) and acetylated α-tubulin (blue) in the mouse organ of corti **(A**–**F)** and the vestibular epithelium **(G**–**L)**. **(A**–**C)** TULP3 immunoreactivity is present in cochlear hair cell kinocilia of IHCs and OHCs (arrowhead). **(D**–**F)** At P8, TULP3 is absent in kinocilia, but localized to the microtubule bundles of Deiters‘cells (DC) and pillar cells (PC) (arrows). **(G**–**L)** In the vestibular epithelium, TULP3 immunoreactivity is present in hair cell kinocilia at P0 (arrowhead) but absent at P8. Scale Bar 5 μm.

At early postnatal stage (P0), TULP3 selectively localized to cilia of the cochlear epithelium (Figures 3A–C). Different from tubby, however, this localization was specific for hair cell kinocilia, whereas TULP3 immunoreactivity was absent from the primary cilia of the supporting cells interspersed with the hair cells in the sensory epithelium. TULP3 was lost from hair cell kinocilia during subsequent early postnatal development. Thus, TULP3 immunoreactivity was not detected at the surface of hair cells by postnatal day 8 (Figures 3D–F).

Given that kinocilia of cochlear hair cells degrade towards the onset of hearing (between P8 and P11; Wang and Zhou, 2021), postnatal loss of TULP3 may be related to the subsequent loss of ciliary function in these hair cells. We were therefore interested in TULP3 expression and localization in vestibular hair cells, where the kinocilium persists as a component of the hair bundle throughout lifetime (Jones and Chen, 2008). As shown in Figures 3G–I, in the vestibular sensory epithelium of P0 mice TULP3 selectively localized to hair cell kinocilia, recapitulating the cochlear postnatal pattern. At later postnatal stages (P8), TULP3 immunoreactivity was no longer detectable in vestibular hair cells, indicating the loss of TULP3 protein from kinocilia similar to cochlear hair cells (Figures 3J–L).

### 3.3. Localization of TULP3 to non-ciliary microtubular structures

**Figure 4:**
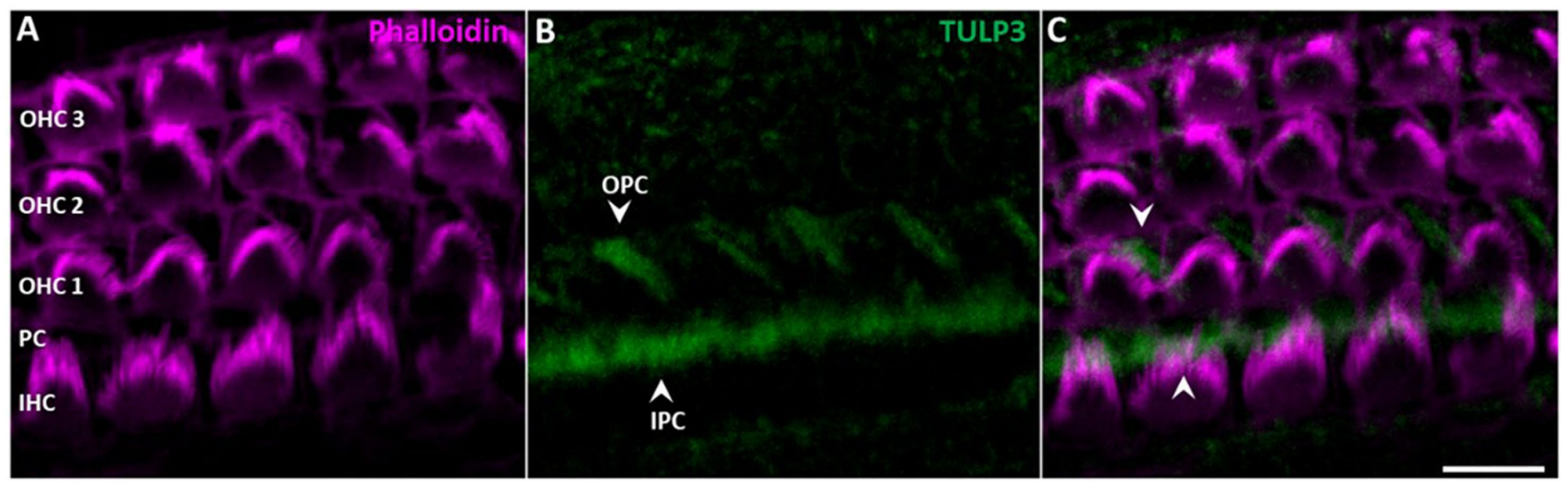
TULP3 immunoreactivity in the cochlear epithelium of P3 mice. **(A**–**C)** P3 whole-mount immunohistochemical staining of TULP3 (green) and Phalloidin (staining actin, magenta) in the mouse organ of corti. TULP3 immunoreactivity in the outer (OPC) and inner pillar cells (IPC, arrowheads) in the Scale Bar 10 μm.

**Figure 5:**
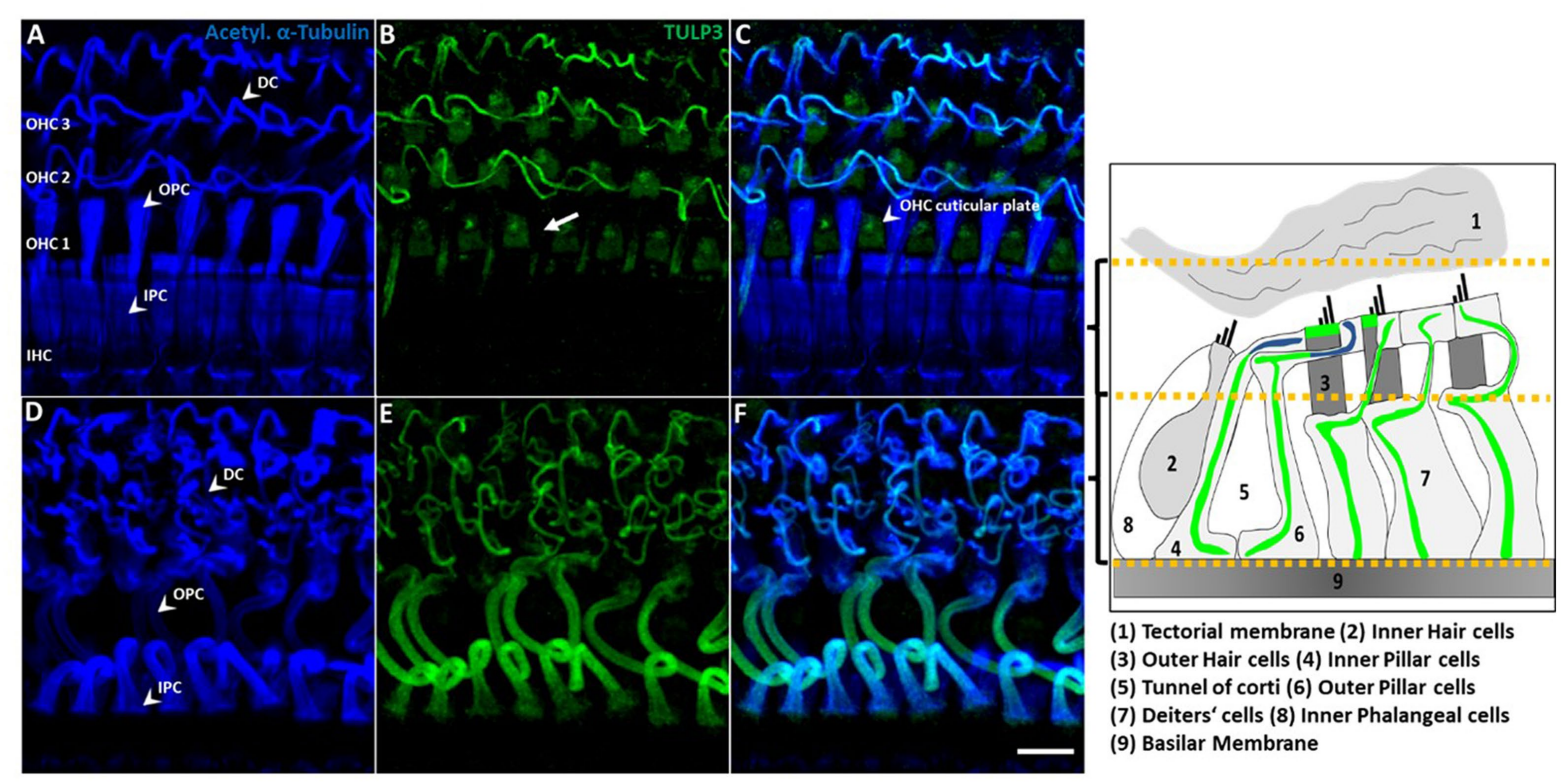
TULP3 immunoreactivity is localized to supporting cell microtubule bundles and the OHC cuticular plate. **(A**–**F)** P30 whole-mount immunohistochemical staining of TULP3 (green) with acetylated α-tubulin (blue) in the mouse organ of corti. TULP3 co-localizes with acetylated α-tubulin in Deiters‘cells (DC), outer (OPC) and inner pillar cells (IPC) but is absent in pillar heads (**B**, arrow). Additionally, TULP3 immunoreactivity is present in the OHC cuticular plate (arrowhead, **C**). Scale bar 10 μm. Right cartoon: Schematic simplification of the detected TULP3 immunosignal within the apical and basal half of the organ of Corti.

While TULP3 was lost from kinocilia during postnatal development, TULP3 immunolabeling progressively appeared in cell bodies of supporting cells (Figures 3, 4). Weak TULP3 signals were detected as early as P3 in outer pillar cells (OPCs) and inner pillar cells (IPC) at the subapical level (Figure 4). This TULP3 staining pattern became successively more pronounced during the postnatal development and additionally well-defined filamentous TULP3-positive structures emerged in the OHC region at this age (Figure 5).

Supporting cells including pillar cells and Deiters’ cells (DCs’) provide structural support to the auditory epithelium by virtue of highly specialized microtubule-rich structures (Liu et al., 2018; Juergens et al., 2020; Chen et al., 2021). Given the known association of TULP proteins with tubulin-based ciliary structures, we wondered whether the observed TULP3-positive non-ciliary structures might correspond to microtubule bundles in the basolateral domain of non-sensory supporting cells. To this end, we co-stained for TULP3 and acetylated α-tubulin in organs of Corti from P30 mice. At P30, TULP3 extensively colocalized with the long pillar core bundles spanning the entire length of IPCs and OPCs and their phalangeal processes underlying the apical cell surface, while it is absent from pillar heads (Figures 5A–F, B, arrow).

Based on co-localization with tubulin, the filamentous TULP3-positive structures between OHCs first observed at P8 (Figure 3) are identified as the DCs’ phalangeal processes. At P30, TULP3 immunoreactivity strongly associated with the microtubule bundles spanning the DCs’ processes (Figures 5B–E). Additionally, some TULP3 immunolabeling was apparent in the OHC cuticular plate, while it was not abundant in IHCs (Figures 5A–C).

## 4. Discussion

Immunolocalization of tubby to the tips of the OHC’s stereociliary bundle confirms previous findings (Han et al., 2020). Thus, the localization is consistent with a role in organizing the protein complexes containing stereocilin, otogelin and otogelin-like (Han et al., 2020; Youn et al., 2022) that form the horizontal top connectors (HTCs) and tectorial membrane-attachment crowns (TM-ACs), required for connection between stereocilia and with the tectorial membrane, respectively (Avan et al., 2019). Previously well-characterized roles of tubby in other cell types relate to the function of primary cilia: tubby mediates delivery of ciliary cargo, such as GPCRs, into neuronal cilia (Sun et al., 2012; Wang et al., 2018; Hong et al., 2021). Thus, in the inner ear tubby has acquired a non-canonical and highly specific function, which can explain the auditory phenotype of tubby-deficient mice. In contrast, retinal and neuroendocrine pathologies of the tubby mouse are ciliopathies, consistent with tubby’s canonical function (Mukhopadhyay and Jackson, 2011; Sun et al., 2012).

**Figure 6:**
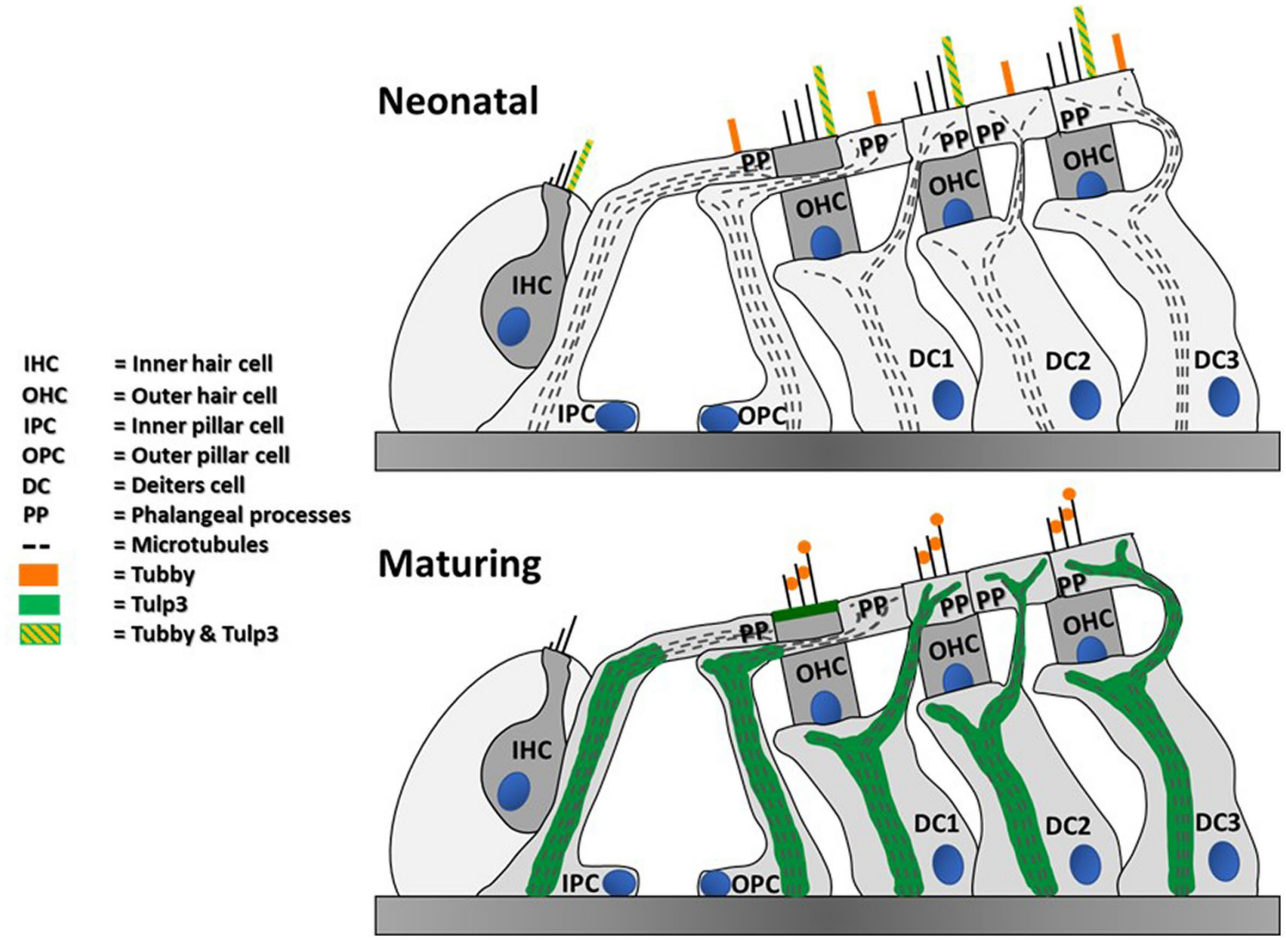
Summary of detected tubby & TULP3 immunoreactivity in the postnatal mouse cochlea. Blue circles represent nuclei.

Interestingly, we additionally detected tubby in hair cell kinocilia and primary cilia of supporting cells during postnatal development, which to our knowledge has not been observed previously. Moreover, we also observed that the tubby homolog, TULP3, is present in the kinocilia of neonatal auditory and vestibular hair cells. Like tubby, TULP3 acts as an adaptor in ciliary trafficking. Both proteins share a common mechanism of action and partially overlap in their cargo spectra (Mukhopadhyay et al., 2010, 2013; Badgandi et al., 2017). Assuming conserved functions across cell types, the roles of tubby and TULP3 in kinocilia of immature hair cells during development may relate to their canonical function, i.e., ciliary trafficking of proteins involved in developmental processes in cochlear and vestibular sensory epithelia. Although potential cargo proteins in hair cells remain to be explored, ciliary proteins previously shown to rely on TULP3 or tubby for their ciliary localization have established roles in hair cell development, namely SSH or polycystin1 (Steigelman et al., 2011; Chen et al., 2017). The cilia-associated pattern of tubby and TULP3 was developmentally regulated, exhibiting a prominent localization in hair cell kinocilia only at early postnatal stage followed by subsequent loss of both proteins to below detectable levels. The developmental down-regulation could simply be associated with the complete degeneration of kinocilia between P8 and P11. However, since a similar developmental pattern was also observed in postnatal development of vestibular kinocilia (Figure 3), where the kinocilium persists throughout maturity (Ciuman, 2011), the loss of tubby/TULP3 from kinocilia may suggest a dedicated developmental role in the pre- or perinatal period (Figure 6).

Of note, stereocilin, otogelin, and otogelin-like also localize to kinocilia of IHCs and OHCs during early postnatal development as well as stereocilin in vestibular hair cells (Verpy et al., 2011; Han et al., 2020). Thus, tubby precisely colocalizes with these components not only in stereocilia tips but also in immature kinocilia. This observation suggests that functional interaction of tubby with these extracellular linker proteins may be conserved between stereocilia and kinocilia.

However, it was reported that while in OHC stereocilia proper stereocilin localization requires tubby, localization of stereocilin in postnatal kinocilia was unaffected in tubby knockout mice. Further, the presence of stereocilin in the kinocilia of vestibular hair cells does not go along with known tubby expression and localization (Verpy et al., 2011; Han et al., 2020). Here, we show that the tubby homolog, TULP3, is present in the kinocilia of neonatal auditory and vestibular hair cells. We thus hypothesize that tubby and TULP3 are functionally redundant in trafficking or organizing the stereocilin complexes in auditory postnatal kinocilia, but not in OHC stereocilia, where tubby is indispensable for the organization or maintenance of stereocilin-containing complexes (Han et al., 2020; Youn et al., 2022). Vice versa, TULP3, rather than tubby, may serve the same role in vestibular kinocilia.

So far, the auditory phenotype of *TUB* deficient mice has been attributed to impaired hair bundle connectivity of mature OHCs resulting from loss of TM-AC and HTC complexes, while there is no obvious defect linked to hair cell kinocilia (Han et al., 2020, Youn et al., 2022). Nevertheless, proper development and function of the cochlea is tightly linked to the cilia of its epithelial cells. Thus, the kinocilium, equivalent to the hair cell’s primary cilium, has a pivotal role in the correct organization of the stereociliary bundle and planar cell polarity (Deans, 2021). The redundant activity of TULP3 may explain the lack of any obvious kinocilia-related defects in tubby-knockout mice.

Like most epithelial cells, cochlear non-sensory supporting cells contain primary cilia as well, although their function in cochlear development and function is yet poorly studied (Wang and Zhou, 2021). Ciliopathies, where mutations in ciliary genes impact structure and function of primary cilia (Quinlan et al., 2008) involve defects not only in various epithelial organs such as kidney and, lung, but also inner ear sensory epithelia.

Our finding that tubby localizes to supporting cell cilia in the immature cochlea seems to predict a potential cilia defect in the sensory epithelium of tubby-deficient mice, which, however, has not been reported with respect to either hearing function or cochlear structure. Thus, tubby in these cilia is either dispensable for their development and physiology, or its loss results in subtle defects yet to be uncovered. Specifically, tubby loss may be compensated by redundant function of tubby homologs other than TULP3.

Although lost from cilia, prominent TULP3 immunolabeling of non-ciliary intracellular structures within cochlear non-sensory supporting cells was observed from P3 to maturity (Figures 3–5). Here, TULP3 co-localized with the pronounced microtubule bundles in IPCs, OPCs and DCs (Slepecky et al., 1995; Zhao et al., 2008). During postnatal maturation of the organ of Corti massive changes in structure and mechanics of supporting cells take place, that involve formation and reorganization of microtubule-rich structures, such as elongation and shape changes of OPCs and IPCs to form the tunnel of Corti, and formation of stiff phalangeal processes by the DCs (Szarama et al., 2012; Soons et al., 2015). TULP3, colocalized to these developing structures, might act as a regulator of the supporting cell microtubule cytoskeleton.

Future studies will be required to explore such a new role of TULP3 and its role in the inner ear.

## Data availability statement

The original contributions presented in the study are included in the article/Supplementary material, further inquiries can be directed to the corresponding author.

## Ethics statement

The animal study was reviewed and approved by the regional council Gießen.

## Author contributions

LL, DD, DO, and KR wrote the manuscript. DO and KR revised the manuscript and designed and supervised the study. LL and DD performed the experiments and analyzed the data. DD created the figures. All authors contributed to the article and approved the submitted version.

## Conflict of interest

The authors declare that the research was conducted in the absence of any commercial or financial relationships that could be construed as a potential conflict of interest.

## Publisher’s note

All claims expressed in this article are solely those of the authors and do not necessarily represent those of their affiliated organizations, or those of the publisher, the editors and the reviewers. Any product that may be evaluated in this article, or claim that may be made by its manufacturer, is not guaranteed or endorsed by the publisher.

## Footnotes

^1^ https://imagej.net/plugins/coloc-2
