# Supplementary material for: Distribution of ciliary adaptor proteins tubby and TULP3 in the organ of Corti
Source: Front Neurosci. 2023 Apr 18;17:1162937. doi: 10.3389/fnins.2023.1162937 (PMC10151737; doi:10.3389/fnins.2023.1162937)
Supplement: Supplementary file 1 [file Data_Sheet_1.docx]

Supplemental material


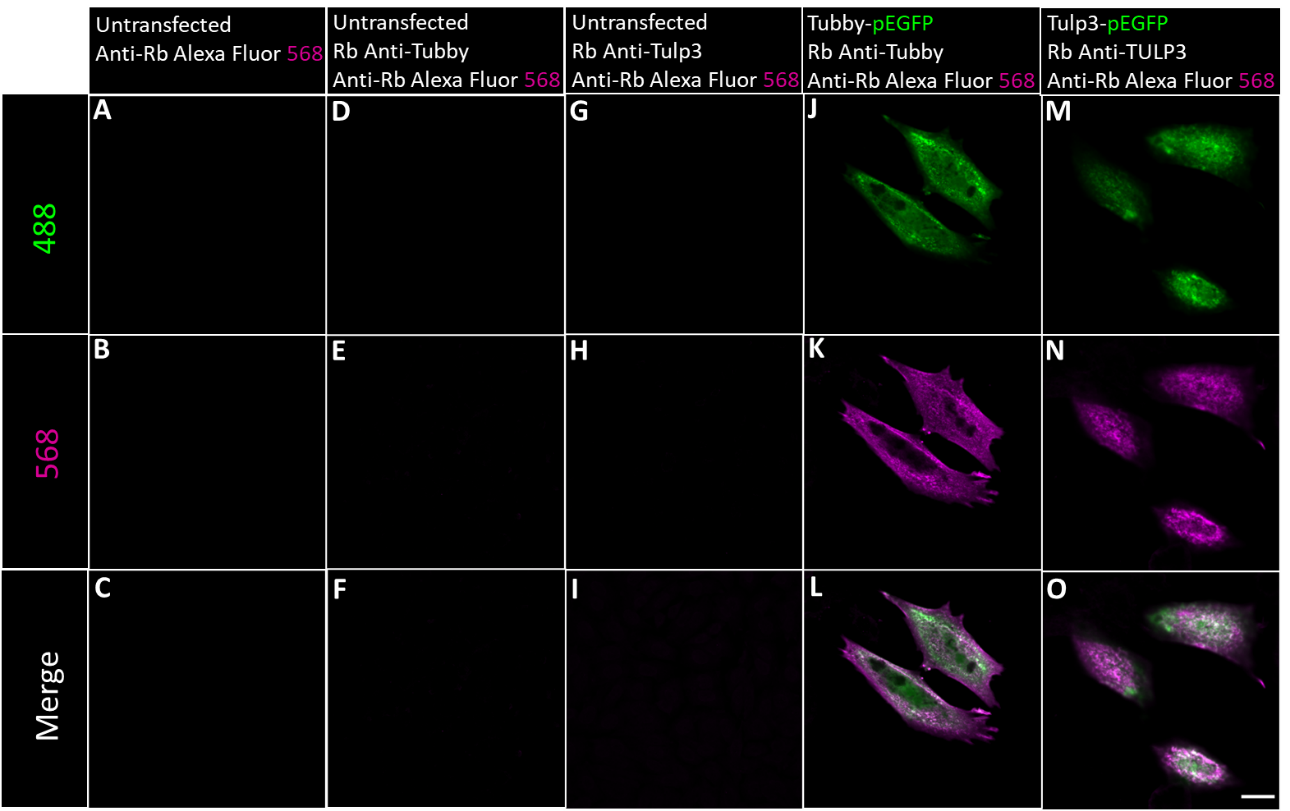


***Supplemental figure 1: Rabbit (Rb) Anti-Tubby and Rb Anti-TULP3 specifically recognize full length EGFP-tagged Tubby and Tulp3 in transfected CHO cells****. (A) - (I) In untransfected CHO cells, neither the secondary antibody only nor primary + secondary antibody show unspecific immunodetection. EGFP-tagged Tubby (green) - and EGFP-tagged TULP3 (green) - positive cells were specifically recognized by Rb Anti-Tubby (J - L) and Rb Anti-Tulp3 (M - O). Scale bar 10 µm.*


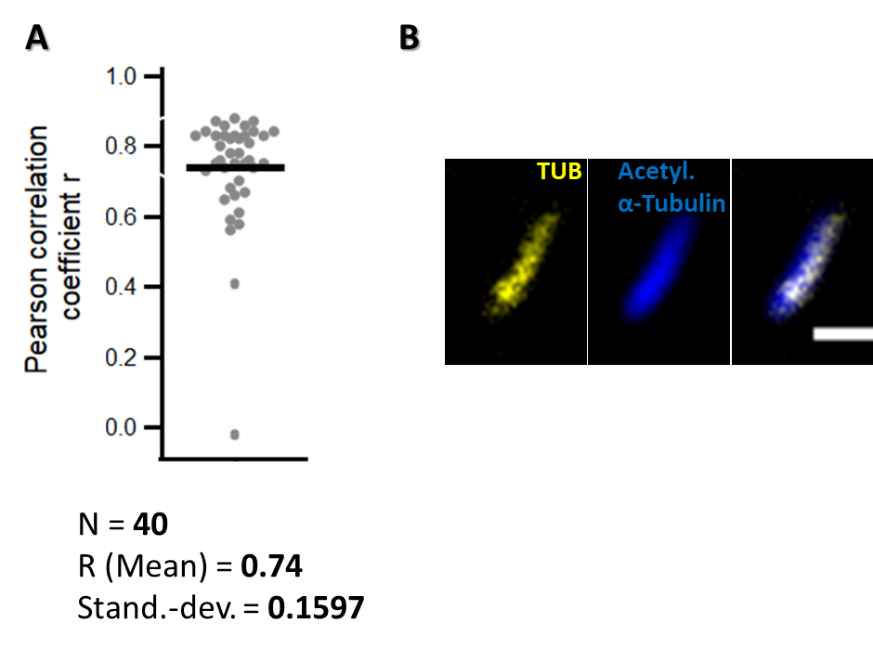


***Supplemental figure 2: Tubby positively correlates with acetylated a-tubulin in P3 mice cochlea.*** *(A) Dot plot of the calculated Pearson’s correlation coefficient. Tubby and acetylated a-tubulin positively correlate with a mean coefficient r = 0.74. (B) Exemplary ROI that was analysed to quantify the colocalization of tubby and acetylated a-tubulin. Scale Bar 1 µm.*
